# Allellic variants in regulatory regions of cyclooxygenase-2: association with advanced colorectal adenoma

**DOI:** 10.1038/sj.bjc.6602806

**Published:** 2005-10-04

**Authors:** I U Ali, B T Luke, M Dean, P Greenwald

**Affiliations:** 1 Division for Cancer Prevention, National Cancer Institute, 6130 Executive Blvd., Bethesda, MD 20892, USA; 2 Center for Cancer Research, National Cancer Institute, Frederick, MD 21702, USA; 3 Advanced Biomedical Computing Center, SAIC, National Cancer Institute, Frederick, MD 21702, USA

**Keywords:** cyclooxygenase-2, colorectal adenomas, polymorphisms, haplotypes

## Abstract

Cyclooxygenase 2 (Cox-2) is upregulated in colorectal adenomas and carcinomas. Polymorphisms in the *Cox-2* gene may influence its function and/or its expression and may modify the protective effect of nonsteroidal anti-inflammatory drugs (NSAIDs), thereby impacting individuals' risk of developing colorectal cancer and response to prevention/intervention strategies. In a nested case–control study, four polymorphisms in the *Cox-2* gene (two in the promoter, −663 insertion/deletion, GT/(GT) and −798 A/G; one in intron 5-5229, T/G; one in 3′untranslated region (UTR)-8494, T/C) were genotyped in 726 cases of colorectal adenomas and 729 age- and gender-matched controls in the prostate, lung, colorectal, and ovarian (PLCO) cancer screening trial. There was no significant association between the *Cox-2* polymorphisms and adenoma development in the overall population. However, in males, the relatively rare heterozygous genotype GT/(GT) at −663 in the promoter and the variant homozygous genotype G/G at intron 5-5229 appeared to have inverse associations (odds ratio (OR)=0.59, confidence interval (CI): 0.34–1.02 and OR=0.48, CI: 0.24–0.99, respectively), whereas the heterozygous genotype T/C at 3′UTR-8494 had a positive association (OR=1.31, CI: 1.01–1.71) with adenoma development. Furthermore, the haplotype carrying the risk-conferring 3′UTR-8494 variant was associated with a 35% increase in the odds for adenoma incidence in males (OR=1.35, CI: 1.07–1.70), but the one with a risk allele at 3′UTR-8494 and a protective allele at intron 5-5229 had no effect on adenoma development (OR=0.85, CI: 0.66–1.09). Gender-related differences in adenoma risk were also noted with tobacco usage and protective effects of NSAIDs. Our analysis underscores the significance of the overall allelic architecture of *Cox-2* as an important determinant for risk assessment.

Upregulation of the inducible isoform of prostaglandin endoperoxide G/H synthase/cyclooxygenase enzyme, PTGS2 or cyclooxygenase 2 (Cox-2), occurs in various cancers (Prescott and Fitzpatrick, 2000). Cyclooxygenase 2 generates multifunctional lipid metabolites, prostaglandins, which are implicated in various cellular processes including proliferation, inflammation, invasion, angiogenesis, and apoptosis (Cao and Prescott, 2002). It is estimated that more than 85% of human colon cancers and 50% of colorectal adenomas have elevated levels of Cox-2 (Eberhart *et al*, 1994).

In mouse models of colorectal carcinoma, disruption of the *Cox-2* gene as well as its selective inhibition resulted in a decreased number of polyps (Oshima *et al*, 1996; Mahmoud *et al*, 1998; Jacoby *et al*, 2000). Nonsteroidal anti-inflammatory drugs (NSAIDs), which inhibit both the constitutively expressed Cox-1 and inducible Cox-2, show a protective effect in numerous epidemiological studies for the incidence of colorectal cancer (Giovannucci *et al*, 1995; Smalley *et al*, 1999). Results from recent randomised clinical trials also demonstrated that aspirin prevented the recurrence of adenoma polyps (Baron *et al*, 2003; Benamouzig *et al*, 2003; Sandler *et al*, 2003). Celecoxib, a selective inhibitor of Cox-2, was shown to have chemopreventive effects in the clinical trial of familial adenomatous polyposis patients (Steinbach *et al*, 2000). Despite considerable heterogeneity in the response of individual patients to celecoxib, there was a significant reduction in the overall polyp burden. Thus, it appears that the expression and activity of Cox-2 have a major role in colon carcinogenesis.

A large number of polymorphisms affecting coding as well as noncoding regions of *Cox-2* have been reported. Genetic variants of *Cox-2* with nonsynonymous single-nucleotide polymorphisms (SNPs) in the coding region may have altered specificity, function, and/or interaction with NSAIDs, and may contribute to colorectal cancer risk. However, the frequency of nonsynonymous SNPs in the *Cox-2* gene, especially in Caucasians, is very low. The expression and/or stability of Cox-2 can also be affected by polymorphisms in the regulatory regions that include the promoter, intronic regions, and the 3′untranslated region (UTR). Genetic variants of *Cox-2* in these regions have been reported previously. A common promoter variant, −765 G/C (rs20417/ss5112606), which is located in the putative Sp1 binding site, was reported to have reduced promoter activity and was associated with reduced plasma C-reactive protein levels with implications for various inflammatory responses (Papafili *et al*, 2002) and with a decreased risk for myocardial infarction and stroke (Cipollone *et al*, 2004). Pima Indians with the variant homozygote genotype at this promoter polymorphism (reported as −899 G/C, rs20417/ss5112606) were found to have a 30% higher prevalence of type II diabetes mellitus (Konheim and Wolford, 2003). Recently, allelic variations in the *Cox-2* gene have been analysed for their association with cancer development. Polymorphisms in the promoter and 3′UTR of the *Cox-2* gene were found to modulate risk for prostate, colorectal, and non-small-cell lung carcinoma (Campa *et al*, 2004; Cox *et al*, 2004; Panguluri *et al*, 2004).

Polymorphisms may also modify the effect of NSAIDs in the prevention of colon cancer and adenomas. Two reports have shown the pharmacological relevance of the polymorphism P17L in the *Cox-1* gene and NSAIDs usage, albeit with opposite associations (Halushka *et al*, 2003; Ulrich *et al*, 2004). There is limited data on the interaction between *Cox-2* SNPs and NSAIDs. More recently, the wild-type and variant genotypes at the −765 position (rs20417/ss5112606) in the promoter region were reported to decrease the risk of colorectal polyp formation in users and nonusers of aspirin/NSAIDs, respectively (Ulrich *et al*, 2005).

In this study, we explored the association between four specific SNPs in the regulatory regions (two in the promoter region (−663 and −798), one in intron 5 (5229), and one in 3′UTR (8494)) and the risk for advanced adenoma in a nested case/control study of 1455 Caucasians (726 cases and 729 controls) from the prostate, lung, colorectal, and ovarian (PLCO) cancer screening trial (Gohagan *et al*, 2000). Furthermore, in view of the strong evidence of tobacco smoking as a risk factor and use of NSAIDs as a protective factor for colorectal cancer, we investigated possible interactions between *Cox-2* gene polymorphisms and the smoking status and use of NSAIDs on the risk of adenoma development.

## MATERIALS AND METHODS

### Study population

The study was conducted using a nested case–control design within the PLCO cancer screening trial, which was designed to evaluate the impact of selected screening procedures on PLCO cancer mortality. The trial recruited approximately 74 000 screening arm participants (37 000 men, 37 000 women; age 55–74 years) and an equal number of nonscreened controls, aged 55–74 years, at 10 US study centres. Participants were randomised to the screening or control arms to evaluate the effect of blood prostate-specific testing and digital rectal examination, chest X-ray, sigmoidoscopy, and trans-vaginal ultrasound and CA-125 testing on PLCO cancer mortality (Gohagan *et al*, 2000). Written informed consent from participants and approval from the institutional review boards of the 10 study centres and National Cancer Institute were obtained. Detailed information on diet, tobacco and alcohol use, intake of selected drugs, family history of cancer, and other risk factors were obtained with a baseline questionnaire.

In this nested case–control study, 726 (506 males and 220 females) individuals with advanced adenomas and 729 (502 males and 227 females) screen-negative gender-matched controls were selected from the screening arm of the trial. The disparity between the number of males and females may partially be due to the initial slow accrual of women as bilateral oophorectomy was originally an exclusion criterion (dropped later) (Weissfeld *et al*, 2005). More importantly, it is a reflection of substantially greater prevalence of large (⩾1.0 cm), intermediate (0.5–0.9 cm), or any size polyp in men than in women; cancer and advanced adenomas were also diagnosed twice as frequently in men in the PLCO trial (Weissfeld *et al*, 2005). Advanced adenoma was defined as villous or tubulovillous adenoma, large adenoma (>1.0 cm), or severe or high-grade dysplasia. The screen-negative controls had no polyp or other suspect lesions. Characteristics of this study population have been described previously (Huang *et al*, 2005). All cases and controls used in this analysis were of Caucasian origin.

### Genotyping

The following four polymorphisms in the *Cox-2* gene, with reasonable frequency distribution in Caucasians, were selected to be analysed for their association with adenoma development: (1) GT insertion/deletion polymorphism at position −663 (positions refer to the Genbank entry AY382629 and http://pga.gs.washington.edu/data/ptgs2/ptgs2.ColorFasta.html) in the promoter with a frequency of deletion: 0.10, (2) A/G polymorphism at position −798 in the promoter with a frequency of G: 0.11, (3) T/G polymorphism at position 5229 in intron 5 with a frequency of G: 0.35, and (4) T/C polymorphism at position 8494 in the 3′UTR with a frequency of C: 0.43. All four polymorphisms were genotyped using the ABI Prism sequence detector (TaqMan; PE Biosystems, Foster City, CA, USA). Polymerase chain reaction primers and dual-labelled allele discrimination probes were designed using the Primer Express software package (PE Biosystems). Oligonucleotide probes were labelled with two different fluorescent dyes, FAM™ and VIC™, to discriminate between the two alleles of the polymorphism. Primer and probe sequences for the four polymorphisms are displayed in Table 1.

The assay was set up in 25 *μ*l reactions with 1–5 ng of genomic DNA and 2 × volume of the Master Mix provided by PE Biosystems, which contains all four deoxynucleotides, *Taq* polymerase and TaqMan buffer, 2000 nm of forward and reverse primers, and the double-labelled probes. The thermal cycling conditions for the ABI prism 7700 Sequence Detector were set up at initial settings of 50°C for 10 min followed by 40 cycles each of 95°C for 15 s and 60°C for 1 min. In each 96-well plate, internal quality controls for homozygous wild-type, heterozygous, and homozygous variant alleles for the respective polymorphism and no template controls were included. Additionally, approximately 10% repeated quality control samples were included. All laboratory personnel were blind to the status of the samples.

### Statistical analysis and haplotype construction

Odds ratios (ORs) were estimated using regression models using PROC LOGISTIC function of the software package SAS (version 8.1; SAS Institute, Cary, NC, USA), adjusting for age, gender, tobacco use, and NSAIDs use. The asymptomatic Pearson's *χ*^2^ test was used to assess departure from Hardy–Weinberg equilibrium by comparing the expected to observed genotype frequencies. All polymorphisms were in Hardy–Weinberg equilibrium, except the one at intron 5-5229. The reasons for this deviation are not clear; however, it is highly unlikely to be due to genotyping errors as the inclusion of the internal and external controls, and the blindedness of the laboratory personnel to the samples ensured accuracy of genotyping results. The possibility that deviation from Hardy–Weinberg at this one position could be due to bias in selection of the controls cannot be ruled out.

Since no homozygous variant at −663 was present and no association was found for the genotypes at −798, haplotypes incorporating only the intron 5-5229 and 3′UTR-8494 genotypes were constructed using the PHASE version 2 software (Stephens *et al*, 2001; Stephens and Donnelly, 2003). Only the haplotypes with known genotypes were used and no inferred haplotypes were included in the analysis.

## RESULTS

### Association of *Cox-2* polymorphisms with adenoma development

Among the subjects analysed in this study, approximately 70% were male. This is consistent with the 2 : 1 ratio of the incidence of colon polyps and tumours in the PLCO trial (Weissfeld *et al*, 2005) as well as reported elsewhere (McCashland *et al*, 2001). The initial assessment of the entire study population detected only trends between certain genotypes and the disease, but no statistically significant associations. We therefore performed association analyses on males and females separately and observed significant gender-based differences. Table 2 displays the association of *Cox-2* genotypes with the risk for advanced adenomas in all subjects and in males and females after standardising for age and smoking history. In males, the heterozygous genotype at −663 with a GT deletion, although infrequent, and the variant homozygotes at the intron 5-5229 positions suggest a decrease in the risk of developing adenomas (OR=0.59, 95% confidence interval (CI): 0.34–1.02 and OR=0.48, 95% CI: 0.24–0.99, respectively). On the other hand, individuals with the heterozygous genotype at 3′UTR-8494 were at an increased risk for developing advanced adenomas (OR=1.31, CI: 1.01–1.71). The OR of 1.02 for the homozygous variant genotype was also in the same direction, but may not have reached significance because of relatively small number compared to the individuals with the heterozygous genotype.

There was no association between the genotypes at any of the four polymorphic sites in the *Cox-2* gene and advanced adenomas in females. However, it is of note that the heterozygous and variant homozygous genotypes at intron 5-5229 showed a trend toward positive association with adenoma risk compared to the suggested protective effect in males. The ORs of genotypes at the −798 and 3′UTR-8494 positions in females, on the other hand, appeared to follow the same trend as in males (Table 2).

The two polymorphisms of the *Cox-2* gene with reasonable frequency and with an association with adenoma development in males, that is, intron 5-5229 and 3′UTR-8494, were used to construct haplotypes using the PHASE program. Three haplotypes, TT, TC, and GC, accounted for 99.6% of all chromosomes. As shown in Table 3, the haplotype TC, which carried the risk allele at the 3′UTR-8494 position, was more common in cases than in controls with a 26% increased risk of adenomas in all subjects (OR=1.26, 95% CI: 1.03–1.53) and with a 35% increased risk of adenomas in males (OR=1.35, 95% CI: 1.07–1.70) compared to the wild-type haplotype of TT. The haplotype was not significant in females. The risk effect of the haplotype TC was neutralised in the haplotype GC, which harboured the protective allele at intron 5-5229 (All, OR=0.95, 95% CI: 0.77–1.16; Males, OR=0.85, 95% CI: 0.66–1.09), showing that the risk of adenoma is determined by the cumulative effects of both polymorphisms. Essentially, the same results were obtained when haplotypes were constructed incorporating all four polymorphisms analysed in this study (data not shown). The effect of the rare deletion polymorphism at −663, which had a negative association with adenomas in males, could not be determined in the context of haplotype as the only haplotype carrying the −663 variant allele was relatively rare.

### *Cox-2* genotypes and tobacco smoking

The use of tobacco is considered to be a risk factor for colorectal cancer. There was a positive association between cigarette smoking and development of aggressive adenomas in the study population, especially a highly significant association between current smokers, both males (OR=2.50, 95% CI: 1.54–4.04) and females (OR=2.51, CI: 1.31–4.80), and risk of colorectal adenoma, when adjusted for age and NSAID use. We assessed the main effect of all four *Cox-2* genotypes on adenoma development when individuals were stratified by smoking status and adjusted for age and NSAIDs usage. Most of the genotypes, especially in current smokers, were associated with a relative increase in the risk of colon cancer compared to never smokers in both males and females (data not shown). Table 4 displays the risk of adenoma development with tobacco usage in the context of haplotypes constructed with intron 5-5229 and 3′UTR-8494 polymorphisms. Compared to never smokers, all three haplotypes had increased risk of adenoma in current female smokers. In current male smokers, carriers of T/T and TC haplotypes were at significantly increased risk of adenoma development, but the ones carrying the haplotype GC with the protective variant at intron 5-5229 did not have a significantly increased risk of adenoma development (OR=1.38, CI: 0.63–3.01). Former male smokers with the haplotype carrying the risk variant at 3′UTR-8494 appeared to be at an increased risk of adenoma development compared to never smokers, whereas the risk for advanced adenoma was not significant in former female smokers with all three haplotypes.

### *Cox-2* genotype and use of NSAIDs

The use of NSAIDs, aspirin, and ibuprofen, which inhibit Cox-2, was not protective for adenoma development in the study population. When analysed at the haplotype level, the use of aspirin was protective for individuals carrying the *Cox-2* haplotype with the wild-type alleles at both intron 5-5229 and 3′UTR-8494 positions (OR=0.74, CI: 0.57–0.95); especially the protection from the use of aspirin in combination with ibuprofen was highly significant (OR=0.55, CI: 0.40–0.77) (Table 5). The use of aspirin appeared to be protective also in male carriers of the haplotype GC, although it did not quite reach statistical significance (OR=0.69, CI: 0.45–1.05). The only striking finding was the use of aspirin in females with the GC haplotype carrying variant alleles at intron 5-5229 and 3′UTR-8494 was associated with increased risk of adenoma development. Although the numbers of cases and controls using aspirin were small, the risk association was statistically significant (OR=2.40, CI: 1.06–5.40).

## DISCUSSION

Analysis of potentially functional polymorphisms in candidate genes has emerged as a powerful approach in deciphering the complex relationship between genotype and phenotype. In this context, association analyses can be used to explore the role of genetic polymorphisms in susceptibility to various cancers and response to specific chemopreventive agents. In our study, analysis of four haplotype-tagging SNPs in the regulatory regions scattered over the entire *Cox-2* gene revealed important aspects of its allelic structure with consequences for adenoma risk and interactions with NSAIDs.

Cox-2 is an inducible enzyme and its expression and stability appear to be subjected to very complex mechanisms regulated by various elements in both the 5′UTR and 3′UTR of the transcript as well as intronic sequences (Dixon *et al*, 2000; Kirtikara *et al*, 2000; Finkbeiner, 2001; Tanabe and Tohnai, 2002; Konheim and Wolford, 2003). Several promoter elements, such as CRE as well as those specific for binding to a variety of nuclear regulatory factors including Nf*κ*B, NF-IL6, and myb, may play an important regulatory role in the transcription of the *Cox-2* gene in a tissue-specific manner (Potter *et al*, 2000; Mestre *et al*, 2001; Tang *et al*, 2001). Polymorphisms may either eliminate or create binding sites for various factors potentially altering the expression of Cox-2 and thereby modulating the risk for various cancers. Recently, four SNPs in the promoter region of *Cox-2* (−297, −899, −1265, −1285), different from the ones used in our study, have been described to modulate the risk for prostate cancer in both African Americans and Caucasian individuals (Panguluri *et al*, 2004).

We examined two promoter polymorphisms, −663 and −798, the *Cox-2* gene which are not located in binding sites of any known transcription factors. The homozygous variant at −663 was extremely rare in our study cohort, but the heterozygous genotype was clearly protective for adenoma risk in males (Table 2). Our data also show that the variant allele at intron 5-5229 reduced the risk for adenoma development in males and, when analysed in the context of haplotype, neutralised the risk effect of the 3′UTR-8494 variant allele. Therefore, either the intron 5-5229 polymorphism itself or another polymorphism that is in linkage disequilibrium with intron 5-5229 in the *Cox-2* gene may be relevant for its transcriptional regulation and/or stability.

Besides the promoter and intronic sequences, elements in the 3′UTR play an important role in polyadenylation, nuclear export, degradation, stabilisation, and translation of the transcripts (Kuersten and Goodwin, 2003). Binding of translational regulatory factors to ARE (AU-rich element) in 3′UTR has been shown to alter Cox-2 expression (Dixon, 2004). *In vitro* experiments have shown that overexpression of trans-activating cellular factors, which bind to *Cox-2* ARE, stabilise mRNA resulting in increased expression of Cox-2 in colon cancer cells (Dixon *et al*, 2000, 2001, 2003). Conceivably, polymorphisms in the 3′UTR of *Cox-2* may modify the binding affinity of regulatory factors and influence its stability and expression. The 3′UTR-8494 polymorphism analysed here is located in the AU-rich region that mediates transcript degradation. Our finding that the 8494 variant allele increases the risk for colorectal adenomas in males by 31% suggests that the polymorphism has a transcript-stabilising function. Interestingly, the same 3′UTR-8494 polymorphism was recently reported to carry an enhanced risk for lung cancer (Campa *et al*, 2004), suggesting its significance in the regulation of *Cox-2* transcripts and the subsequent impact on multiple cancers. However, the same polymorphism did not show an effect on colorectal cancer in another recent report, although two downstream SNPs were associated with an increased risk (Cox *et al*, 2004). This lack of association of the 3′UTR-8494 polymorphism with colorectal cancer risk (Cox *et al*, 2004) could be due to relatively small number of subjects (292 cases/274 controls) and/or analysing males and females together. In any event, association of the 3′UTR-8494 variant and/or nearby polymorphisms in colorectal and lung carcinogenesis warrant a closer look at this region for its role in post-transcriptional regulation of the *Cox-2* gene with implications for colorectal and lung carcinogenesis.

In our study, sequence variations in the *Cox-2* gene, with a potential to affect its expression and/or stability, appear to modulate the risk for adenoma development only in males. Previous studies have documented gender differences in the incidence, location, and pathogenesis of colonic adenomatous polyps and tumours (DeCosse *et al*, 1993; McCashland *et al*, 2001; Weissfeld *et al*, 2005). The reduced colorectal cancer risk in women has been attributed to physiological, environmental, and behavioural factors (DeCosse *et al*, 1993). Especially, oestrogen has been suggested to have a protective role in colon carcinogenesis by affecting metabolism of bile acids and serum levels of insulin-like growth hormone levels (Everson *et al*, 1991; Campagnoli *et al*, 1993). Transcriptional silencing of the oestrogen receptor gene has been reported in colorectal tumours (Issa *et al*, 1994) and a meta-analysis of several epidemiological studies of postmenopausal women reported a significant reduction in the risk of colorectal cancers with hormone therapy (Grodstein *et al*, 1999). The apparent lack of any role of *Cox-2* polymorphisms in the development of adenomas in females noted in our study could simply be due to relatively small numbers analysed and certainly needs further validation. A comprehensive analysis of various Cox-2 haplotypes in a much larger case–control study comprising of females would help clarify this issue.

Another important aspect of our analysis is that it highlights the significance of the overall allelic architecture of *Cox-2* on disease risk rather than its effect based on individual SNPs in isolation. Among the four polymorphisms analysed, the combination and/or interplay between two SNPs (minor allele frequencies of 0.35 and 0.43 for intron 5-5229 and 3′UTR-8494, respectively) were important in determining the risk of developing colorectal adenomas. The allelic influence of these two individual polymorphisms conferring either risk of or protection from adenoma development was neutralised in individuals carrying both variants (Table 3).

Cyclooxygenase 2 is a primary target for NSAIDs, which have been shown to reduce the risk of colon cancers and colorectal adenomas. Aspirin use was associated with a decrease in the recurrence of colorectal adenomas in randomised clinical trials (Baron *et al*, 2003; Benamouzig *et al*, 2003; Sandler *et al*, 2003). Sequence variations in the *Cox-2* gene could conceivably modify the chemopreventive effect of aspirin (Ulrich *et al*, 2005) and possibly other NSAIDs. In our analysis, the *Cox-2* haplotype with wild-type alleles of intron 5-5229 and 3′UTR-8494 (also of −663 and −798, data not shown) was pharmacologically beneficial in males (Table 5). The GC haplotype carrying the protective and risk alleles was also suggestive of a beneficial effect in male aspirin users. On the contrary, not only that females do not appear to drive any benefit from aspirin use, female carriers of the GC haplotype were at a significantly increased risk for adenoma development. Of cautionary note here is, the increased risk, although significant, was based on small number of female aspirin users with the GC haplotype.

The gender difference has also recently been reported in the cardiovascular response to aspirin. The risk of myocardial infarction or death from cardiovascular causes in women was not altered by aspirin but was significantly reduced in men (Steering Committee of the Physicians' Health Study Research Group, 1989; Levin, 2005; Ridker *et al*, 2005). Our preliminary observation of the harmful effect of aspirin on colorectal adenoma in females, if confirmed in a larger study, would be helpful in pharmacological stratification of patients according to the *Cox-2* haplotypes.

Cyclooxygenase 2 is a crucial enzyme in a key signalling pathway and has been the target of prevention/intervention strategies in many clinical trials. Selective inhibition of Cox-2 results in variable responses in individual patients. An exhaustive approach using the haplotype-defining SNPs in the gene as well as information on the functional significance of polymorphisms with the risk-modulating ability would have significant implications not only for risk identification but also for pharmacological management of the disease.

## Figures and Tables

**Table 1:** Polymorphisms, primers, and probes

**Polymorphism**	**dbSNP**	**Forward primer**	**Reverse primer**	**FAM™ probe**	**VIC™ probe**
–663 GT (insertion/deletion)	rs68946	aggacttaggacataactg aattttctatt	ggagcatgtcagggtga gatact	ttttctggtgtgtgtgtat	ttcttttctggtgtgtgtata
–798 A → G	rs68946	catgatagatgttaaataa	tggaacatagttggatga aagcaaaga	aaattccagctgtcaaa ggaattaat	aattccaactgtcaaaat
5229 T → G	rs20432	ttttataaataatacattt	aacggaattaatatactat attgagcttatatcatata	atacttttttagaattac catatc	atacttttttataattacca tatca
8494 T → C	rs5275	catcttccatgatgcatta gaagtaa	aataatgcactgatacct gtttt	tgacagaaaaataac caaaa	aaaatgaccaaaagtac

dbSNP, single nucleotide polymorphism database. Numbers refer to positions of polymorphisms in the Genbank entry AY382629 and as detailed at http://pga.gs.washington.edu/data/ptgs2/ptgs2.ColorFasta.html.

**Table 2:** *Cox-2* genotypes and risk of advanced colorectal adenomas

**All**			**Males**		**Females**	
**Genotypes**	**Cases/controls**	**OR** **95% CI**	**Cases/controls**	**OR** **95% CI**	**Cases/controls**	**OR** **95% CI**
−*663*						
GT/GT	702/704		490/478		212/226	
GT/(GT)^a^	32/45	0.65 (0.40, 1.04)	24/36	0.59 (0.34, 1.02)	8/9	0.95 (0.35, 2.5)
						
−*798*						
A/A	490/493		339/340		151/153	
A/G	233/231	1.017 (0.81, 1.27)	167/158	1.06 (0.81, 1.39)	66/73	0.91 (0.60, 1.37)
G/G	18/26	0.80 (0.43, 1.50)	11/16	0.79 (0.35, 1.76)	7/10	0.82 (0.30, 2.27)
						
*5229*						
T/T	534/530		384/363		150/167	
T/G	189/190	0.94 (0.74, 1.20)	125/130	0.88 (0.66, 1.18)	64/60	1.15 (0.75, 1.77)
G/G	24/33	0.73 (0.42, 1.27)	13/23	0.48 (0.24, 0.99)	11/10	1.43 (0.58, 3.55)
						
*8494*						
T/T	311/347		215/244		96/103	
T/C	344/318	1.17 (0.94, 1.46)	249/211	1.31 (1.01, 1.71)	95/107	0.94 (0.63, 1.41)
C/C	94/91	1.14 (0.82, 1.59)	59/64	1.02 (0.68, 1.53)	35/27	1.40 (0.78, 2.53)

OR=odds ratio; CI=confidence interval. Adjusted for age and smoking history.

a Parentheses refers to deletion polymorphism.

**Table 3:** Haplotype frequencies constructed by PHASE algorithm in cases and controls, adjusted for age and smoking history

**Haplotype**	**All**		**Males**		**Females**	
**Intron 5-5229 and 3′UTR-8494**	**Cases/controls**	**OR** **95% CI**	**Cases/controls**	**OR** **95% CI**	**Cases/controls**	**OR** **95% CI**
TT	959/1006		676/694		283/312	
TC	298/247	1.26 (1.03, 1.53)	217/165	1.35 (1.07, 1.70)	81/82	1.06 (0.74, 1.51)
GC	234/253	0.950 (0.77, 1.16)	150/174	0.85 (0.66, 1.09)	84/79	1.200 (0.84, 1.71)

OR=odds ratio; CI=confidence interval. Adjusted for age and smoking history. Almost identical results were obtained using the PL-EM and SNPHAP algorithms.

**Table 4:** *Cox-2* haplotypes and risk for advanced colorectal adenomas in association with tobacco usage

**Haplotype**	**Never smoker**		**Current smoker**		**Former smoker**	
**Intron 5-5229 and &3′UTR-8494**	**Cases/controls**	**OR** **95% CI**	**Cases/controls**	**OR** **95% CI**	**Cases/controls**	**OR** **95% CI**
*All*						
TT	377/458		142/63	3.12 (2.24, 4.36)	440/485	1.13 (0.93, 1.38)
TC	111/109	1.24 (0.92, 1.68)	42/16	3.71 (2.03, 6.77)	145/122	1.50 (1.13, 1.99)
GC	89/124	0.86 (0.63, 1.17)	25/18	1.83 (0.97, 3.45)	120/111	1.33 (0.99, 1.79)
						
*Males*						
TT	228/274		97/49	2.81 (1.89, 4.18)	351/371	1.20 (0.95, 1.52)
TC	76/64	1.41 (0.96, 2.07)	28/11	3.76 (1.80, 7.83)	113/90	1.64 (1.17, 2.29)
GC	47/73	0.74 (0.49, 1.12)	15/13	1.38 (0.63, 3.01)	88/88	1.28 (0.90, 1.82)
						
*Females*						
TT	149/184		45/14	4.45 (2.32, 8.51)	89/114	1.00 (0.70, 1.43)
TC	35/45	0.99 (0.60, 1.64)	14/5	3.82 (1.32, 11.0)	32/32	1.25 (0.72, 2.15)
GC	42/51	1.05 (0.65, 1.68)	10/5	3.10 (1.01, 9.48)	32/23	1.73 (0.96, 3.12)

OR=odds ratio; CI=confidence interval. Adjusted for age and NSAIDs usage.

**Table 5:** *Cox-2* haplotypes and risk for advanced colorectal adenomas in association with NSAIDs use

**Haplotype**	**None**		**Aspirin**		**Ibuprofen**		**Both**	
**Intron 5-5229 and 3′UTR-8494**	**Cases/controls**	**OR** **95% CI**	**Cases/controls**	**OR** **95% CI**	**Cases/controls**	**OR** **95% CI**	**Cases/controls**	**OR** **95% CI**
*All*								
TT	422/397		301/302	0.87 (0.70, 1.07)	106/121	0.83 (0.61, 1.12)	132/186	0.65 (0.50, 0.85)
TC	123/102	1.11 (0.82, 1.5)	101/68	1.30 (0.92, 1.82)	34/31	1.03 (0.62, 1.73)	40/46	0.81 (0.51, 1.27)
GC	97/111	0.77 (0.56, 1.0)	73/67	0.92 (0.61, 1.32)	25/34	0.69 (0.404, 1.195)	39/41	0.93 (0.58, 1.48)
								
*Males*								
TT	309/266		222/236	0.742 (0.57, 0.95)	61/67	0.78 (0.52, 1.15)	84/125	0.55 (0.40, 0.77)
TC	91/64	1.18 (0.82, 1.7)	77/52	1.17 (0.79, 1.74)	20/16	1.09 (0.55, 2.18)	29/33	0.75 (0.44, 1.28)
GC	60/74	0.64 (0.44, 0.9)	53/57	0.69 (0.45, 1.05)	13/20	0.59 (0.28, 1.22)	24/23	0.92 (0.50, 1.69)
								
*Females*								
TT	113/131		79/66	1.32 (0.87, 2.00)	45/54	0.98 (0.610, 1.583)	48/61	0.90 (0.57, 1.43)
TC	32/38	0.96 (0.55, 1.6)	24/16	1.66 (0.83, 3.31)	14/15	1.04 (0.47, 2.28)	11/13	0.93 (0.39, 2.20)
GC	37/37	1.11 (0.65, 1.8)	20/10	2.40 (1.06, 5.40)	12/14	0.90 (0.39, 2.06)	15/18	1.01 (0.48, 2.13)

OR=odds ratio; CI=confidence interval. Adjusted for age and smoking history.
